# Case Department

**Published:** 1886-10

**Authors:** 


### CASE DEPARTMENT.

Three cases of general Sarcoma in the horse. Pathological Institute of the Royal Veterinary School in Berlin. Reported by A. Stickes, Assistant to the Professor. Translated from the manuscript by A. W. Clement, V. S.

CASE NO. 1.—A hard worked horse was admitted to the hospital for what was supposed to be a severe attack of colic: death in six hours.

*Autopsy.* Well nourished cadaver. Abdomen much distended: rigor-mortis, strong. The abdominal cavity contained about ten quarts of dark bloody fluid. The duodenum for a short distance from the beginning of the illness infiltrated with blood, as was also the ligamentum recto-duodenalis and that part of the rectum connected with this ligament. In the region of the left kidney there was a soft dark red mass about the size of a child's head, in part covered by and in part protruding through a rupture of the peritoneum: this mass consisted of a clump of dark clotted blood mixed with the fat tissue surrounding the kidney. On the inner surface of this mass were many small tumors, varying in size from a hazelnut to that of a walnut, and of a greenish red color. The renal artery and vein were intact, and the supra-renal capsules normal. The blood-vessels supplying the fibrous capsule of the kidney were much enlarged and in many places perforated; the coagula in the neighborhood of these perforations contained broken down tumor substances. The left kidney measured 25 cm. in length and 12 cm. in width.

The tunica propria slightly thickened, and in many places projecting from the under surface were tumors about the size of a walnut, which pressed upon the substance of the kidney. The surface of the kidney was dark red in color and roughened. On section the cortex was dark red in color, but the histological character could not be made out on account of decomposition ; the medullary portion was bright red in color and the tubules were plainly visible. The right kidney measured 25 cm. in length, and 15.5 cm. in width ; the surface grayish-brown in color. On section the cortex was seen to be yellowish-red in color ; the tubules were plainly visible, the glomeoule not visible. The medullary portion was bright yellow in color, and a yellowish-white fluid could be pressed out of the papillae.

The heart was enlarged ; measured from base to apex, 22 cm., left ventricle 3.5 cm. thick, right ventricle 1.5 cm. thick. The viceral surface of the pericardium was transparent, the muscle substance gray, anaemic and very fragile. The bicuspid valves considerably, the artic valves but slightly thickened. The valves of the right side of heart normal. The endocardium of the left ventricle was slightly swollen ; beneath the endocardium in the musculi papillariae, the trabeculae, and on the attached borders of the bicuspid valves, many bodies, some white and some red, varying in size from a pin's head to a pea, were to be seen. In the right ventricle only hemorrhagic points were to be seen.

*Histology.* The swollen masses consisted of large spindle shaped cells with very little inter-cellular substance, but very vascular. The smaller nodules showed the same cellular structure.

CASE NO. 2.—A chestnut gelding presented the following external lesions. General scleroderma, multiple abscesses, and swelling of the lymph glands.

Clinical diagnosis; chronic glanders. The autopsy made a few hours after death showed the following lesions : the skin was about double its normal thickness, firmly bound to the body, and in many parts covered with a dirty gray scurvy. The subcutis, especially of the extremities presented a firm fibrous appearance ½ to 2 cm. thick, with here and there small yellow decayed masses. The lymph vessels were greatly swollen and contained a large quantity of clear amber colored fluid. The lymphatic glands, especially in the inguinal and axillary regions, were very much enlarged and on section presented a grayish-white clouded appearance. They were of firm consistence. A large quantity of clear scum was found in the articula cavities and in the sheaths of the tendons. Small aggregated masses of firm white bodies, varying in size from a pin's head to about three times that size, were found under the serosa of the stomach. Spleen much enlarged : measured 49 cm. in length and 4.5 cm. in thickness ; surface, steel blue ; under the capsule of the unattached portion were three, large, white, firm, nodules, in the neighborhood of which the blood-vessels were very prominent. The lymph glands of the spleen were as large as a dove's egg, firm, and when cut through seem to be infiltrated with granular whitish-gray bodies about the size of a millet seed, and very closely packed together. The liver was enlarged and firm ; under the capsule were single, dispersed, coarse, white grains ; in the porta hepatis these grains were closely packed together.

The lymph glands of the liver were enlarged to the size of a man's head, firm, and on section finely granular. On the peritoneal surface of the diaphragm, in the neighborhood of the columnar vertebralis, were many firm bodies the size of a millet seed. These same bodies were to be seen on the plueral surface of both lungs in the diophragmatic region. The lung parenchyma was crepitant throughout and bright red in color. The bronchial, intestinal and inferior tracheal glands were much swollen, firm, and on section, grayish white and granular. On the superior border of the trachea closely adherent to the cartilaginous rings were firm swollen masses about the size of a hazelnut, which on section showed the peripheral portion firm and rough, the centrum yellowish-white and decayed. Many milliary bodies covered the pericardium. The heart was much enlarged, weighed 9000 grams ; the left verticle measured 24 cm. in length, the right ventricle 21 cm. The right auricle proper was swollen to the size of a child's head, and covered with tumors the size of a walnut, which were closely set together. Under the epicardial surface of the right auricle were many white bodies the size of a millet seed, closely crowded together and covering the entire surface of the auricle ; this same condition existed on the surface of both ventricles. Under the endocardium, especially on the septal surface of the left ventricle and on the bicuspid valves, were these white millet seed sized bodies likewise to be seen, while the cavity of the right auricle was nearly filled with one mass of tumor. The nasal mucous membrane was in many parts thickened and opaque, especially in posterior part of the concha media the membrane was covered with thick mucous. Extending back from the posterior part of the concho media to the posterior nares, beneath the membrane, were many cloudy submilliary bodies. In the ductus nasalis media were many caseated milliary bodies. Small yellow masses were also to be seen in the introcostal muscles. The histology both of the larger masses and the smaller nodules was the same throughout ; spindle shaped cells with fibrous intercellular substance. Chronologically arranged, perhaps the pathological changes occurred in the following way : Peritonitis, plueritis, pericarditis sarcomatosa ; by metastasis the spleen muscles, myocardium, diaphragm, stomach cartilages of the trachea, and mucous membrane of the nose. Lymph-admitis sarcomatosa of the retro-peritoneal glands of the liver and spleen, the mediostival and bronchial glands. Chronic processes of the sub-cutaneous tissue, and of the lymph glands and vessels of the extremities. Rhinitis catarrahlis chronica.

CASE NO. 3.—The body of a ten-year-old gelding was sent to the institute with the following history : Admitted three days before with symptoms of thoracic disease ; dullness on percussion over the lower third of right side of thorac; on auscultation, bronchial breathing could be heard; respirations greatly increased in frequency; death apparently from dyspnœa; diagnosis, pleuro-pneumonia-dextra.

*Autopsy*—The abdominal cavity contained a large quantity of dark, red fluid. The organs were in their normal position; the mucous membrane of right-half stomach was greenish-white in color, the rugae very prominent and in many places there was erosion of the epithelium. The mucous membrane of the pyloric portion was swollen, cloudy, and covered with firmly adherent mucous; the mucous membrane of the small intestine was grayish-white in color, and the follicles visible to the naked eye. The cæcum and colon were tympanitic, the mucous membrane red and in some parts greenish. The rectum was throughout its entire length, filled with faeces and distended to about the size of a man's arm except at its very commencement; at this part were two circumscribed masses, situated directly opposite each other, just beneath the serosa, causing a bulging on the outside and stenosis of the lumen.

At the end of the rectum, very near the anus, was a swollen mass 26 cm. in length, made up of tumors varying in size from a hen's egg to an apple, round and firm. The spleen weighed 1,100 grams; the capsule was a little thickened and firm in consistence; on section the trabecular were plainly visible, the pulp firm and brownish-red in color. The two kidneys weighed 2,500 grams; the capsules easily removed, the medullary substance reddened and the tubules plainly visible; the tubuli recti and tubuli contorti enlarged, cloudy and grayish-white in color; the glomeruli were injected. The liver weighed 6,500 grams; the capsule thin and soft; in the middle lobe were many calcified bodies about the size of millet seeds, and for the most part arranged in an orderly manner; the parenchyma was firm, and brownish-red in color.

Projecting from the peritoneum, covering the upper surface of the xiphoid cartilage, was a mass of tumor about the size of a child's head, which weighed 2,000 grams, lobed in appearance and lobular on section. The heart weighed 3,000 grams; no structural change. The pleural cavity contained no fluid; the pleura-costalis and pleura-diaphragmatica were covered with processes about 4 cm. long which had the appearance of mucous. On the posterior border of the right lung was a swollen mass about one-half the size of a horse's head, weighing 6,500 grams, lobular in character; each lobule seen on section to vary in size from a dove's egg to a hen's egg; there was a very considerable interlobular tissue rich in blood-vessels; these-blood vessels could be seen in places to pierce the lobules. Single lobules looked white with a yellowish shade, soft, without degeneration, about the consistence of brain matter. The blood-vessels, on leaving this mass, passed over the surface of the lung under the pleura and were distended to the size of a lead pencil, and filled with clotted blood. These vessels could be traced directly to a swollen mass in the mediastinum, which weighed 7,200 grams.

The œsophagus lay upon this swollen mass, and its walls were 1 cm. thick. The bronchial and mediaspinal lymph glands were firm and bluish-red in color ; the intersticial tissue hypertrophied, the parenchyma atrophied. Both lungs were strongly retracted. On cross section glistening, dark red in color, and non crepitant, except in the anterior lobe. On microscopic examination of the mass from the rectum, one can see that the sarcoma starts from the blood-vessels, the walls of which were infiltrated with spindle cells, with oval nuclei, to such an extent as to render the normal tissue unrecognizable. The cells of the whole mass very long and arranged in bands. No intercellular tissue could be made out.

Diagnosis: Sarcoma of the rectum; metastasis to peritoneum, pleura, etc.

*Conclusions.* These three cases present all the same characteristics ; the metastasis took place by means of the blood-vessels and not by the lymph vessels. In case No. 1 the retro peritoneal sarcoma was the primary tumor. In the greatly dilated vessels of the capsule of the kidneys the walls were found to be completely infiltrated with sarcoma cells. A piece had probably become detached and through the circulation the infectious material was carried to the right heart, then through the lung to the left ventricle and by the left coronary artery to the wall of the left ventricle, where many milliary sub endocardial tumors were to be seen. There was no affection of the lymph glands. In case No. 2 there was also no affection of the lymph glands, though there was general sarcoma throughout the abdomen and thorax. The sarcoma in the mediastinum was closely connected with that of the lung and heart, as shown by the greatly dilated condition of the blood-vessels between these parts. The mediostinal and bronchial lymph glands were *not* involved in the sarcomatous process ; they showed simply hypertrophy of the introcellular tissue and atrophy of the parenchyma. In case No. 3, where the greatest extent of tumor infection was found, it is harder to draw conclusions. Yet I think that even here I make no mistake in saying that the sarcomatous infection of the lymph glands was secondary.

"It is a characteristic feature of sarcoma," says Billroth, "that the lymph glands either not at all, or very late, become affected." In this case the heart was the primary seat of infection ; it weighed 9 kilograms or three times what the heart of an ordinary sized horse should weigh, while the tumors in the other organs reached only the size of hazelnuts. Later followed the infection of the lymph glands, which according to the rapidity of growth characteristic of these bodies, soon reached the size of a man's fist. The involvement of all the lymph glands of the abdomen and thorax offered a great obstacle to the circulation of the lymph, and this clearly gave rise to the hypernutrition of the skin, especially of the extremities. Finally it may be said that in all these cases there was no especial cachexia so far as external appearances could show ; the subcutaneous fat tissue was normal ; the hair was smooth and glistening. These cases go to establish the following conclusions, viz ;

1. The secondary infection of sarcoma is carried out by means of the blood-vessels.

2. The affection of the lymph glands follows, if at all, very late.

3. General sarcomatosa progresses, contrary to the opinion formerly held, not necessarily though a special cachexia.

